# Vaccine Designs Utilizing Invariant NKT-Licensed Antigen-Presenting Cells Provide NKT or T Cell Help for B Cell Responses

**DOI:** 10.3389/fimmu.2018.01267

**Published:** 2018-06-04

**Authors:** Shin-ichiro Fujii, Satoru Yamasaki, Yusuke Sato, Kanako Shimizu

**Affiliations:** Laboratory for Immunotherapy, RIKEN Center for Integrative Medical Sciences (IMS), Yokohama, Japan

**Keywords:** NKT, Tfh, NKTfh, dendritic cell, vaccines

## Abstract

Vaccines against a variety of infectious diseases have been developed and tested. Although there have been some notable successes, most are less than optimal or have failed outright. There has been discussion about whether either B cells or dendritic cells (DCs) could be useful for the development of antimicrobial vaccines with the production of high titers of antibodies. Invariant (i)NKT cells have direct antimicrobial effects as well as adjuvant activity, and iNKT-stimulated antigen-presenting cells (APCs) can determine the form of the ensuing humoral and cellular immune responses. In fact, upon activation by ligand, iNKT cells can stimulate both B cells and DCs as *via* either cognate or non-cognate help. iNKT-licensed DCs generate antigen-specific follicular helper CD4^+^ T cells, which in turn stimulate B cells, thus leading to long-term antigen-specific antibody production. Follicular helper iNKT cell-licensed B cells generally produce rapid, but short-term antibody. However, under some conditions in the presence of Th cells, the antibody production can be prolonged. With regards to humoral immunity, the quality and quantity of Ab produced depends on the APC type and the form of the vaccine. In terms of cellular immunity and, in particular, the induction of cytotoxic CD8^+^ T cells, iNKT-licensed DCs show prominent activity. In this review, we discuss differences in iNKT-stimulated APC types and the quality of the ensuing immune response, and also discuss their application in vaccine models to develop successful preventive immunotherapy against infectious diseases.

## Introduction

The success of vaccination strategies against viral infection or cancer depends on the efficient generation of appropriate antigen-specific T and B cell responses. Adequate antibody (Ab) responses of appropriate specificity elicited by vaccination are required to control and protect against many viral pathogens, such as influenza, human immunodeficiency virus, and human papilloma virus (1). Not only the suitable form of antigen, e.g., the commonly used inactivated virus, live attenuated virus, and recombinant viral protein, but also the optimal adjuvant are required for a successful vaccine. The development of ideal vaccine systems has been intensively explored to enhance the efficacy of weak antigens and broaden the immune response profile, leading to generation of high titer broadly neutralizing anti-viral antibodies.

Vaccines targeting B cells are essentially, of two types, T-dependent and T-independent, based on the requirement for T-cell help for Ab production (2). T-independent B cell responses are usually elicited by non-protein antigens that are unable to stimulate Th cells. Multimeric haptens or polysaccharides are typical T-independent B cell antigens that are recognized *via* the B cell antigen receptor (BCR). T-independent antigens generally induce robust and rapid B cell antibody responses, but with a low level of somatic hypermutation and thus affinity maturation, and limited isotype switching. T-dependent responses are typically induced by protein antigens and, as the term implies, there is cognate T-cell help for the antigen-specific B cells (3), which is provided by a specialized subset of CD4^+^ T cells called T follicular helper (Tfh) cells. When antigens contact B cells in the follicles of secondary lymphoid organs, the antigen is internalized by the B cells upon binding to antigen-specific BCRs. The antigen is then processed and antigen-derived peptides are presented in the context of MHC class II (MHC II) molecules. Subsequently, the activated B cells are recruited to the border of the T cell and B cell zones, in which Tfh cells are generated following interacting with dendritic cells (DCs) presenting the same antigen. For the generation of Tfh cells, upregulation of the transcriptional repressor Bcl-6, costimulation by CD28, and stimulation with IL-21 have been reported as important factors (3). Also, by upregulating CXCR5, Tfh cells in turn localize to the boundary of the T and B cell zone (3), which is critical location for B cells to encounter Tfh cells.

Besides these classical T-dependent and T-independent vaccines, NKT cell-mediated vaccines have also been tested as a third vaccine candidate. NKT cells constitute approximately 0.05–0.2% of lymphocytes among human peripheral blood mononuclear cells and are classified into two groups: type I NKT cells express the invariant Vα14-Jα18 TCRα chain paired with either Vβ2, Vβ7, or Vβ8 in mice and Vα24-Jα18/Vβ11 in humans (4). The type I, invariant NKT cells (hereafter iNKT) recognize glycolipids, such as α-GalCer. By contrast, type II NKT cells display more diverse αβ-TCR pairings and respond to sulfatide, but do not to α-GalCer (5). Several reports have shown that iNKT cells can deliver helper signals to B cells directly or indirectly. In infectious diseases, neutralizing Ab production induced by vaccines represents a major protection mechanism against pathogens. Here, we review the features of iNKT cell-mediated Ab production, particularly by interacting directly or indirectly with B cells. We also discuss how these two pathways, i.e., vaccines utilizing iNKT cell help for B cells or iNKT cell help for DCs, augment efficient antigen-specific Ab production in the development of vaccination strategies against infectious diseases.

## The Role of iNKT Cells in Infectious Diseases

Realization of the importance of iNKT cells in protection from infectious diseases has largely been based studies of the responses of Jα18- or CD1d-deficient mice, both of which lack iNKT cells, to viruses, bacteria, and parasites (6, 7). The outcome of most of these infectious models is worse in the iNKT-deficient animals. In studies of viral infections, iNKT cells play a protective role against influenza virus and cytomegalovirus (8, 9), herpes simplex virus type 1, and hepatitis B virus (10). In bacterial infection models, iNKT cells have been shown to be important against *Pseudomonas aeruginosa, Streptococcus pneumoniae, Mycobacterium tuberculosis* (11), *Chlamydia pneumoniae, Sphingomonas paucimobilis*, and *Staphylococcus aureus* (12). The protective responses of iNKT cells during infections are mediated by two mechanisms. First is the direct activation by stimulation of the NKT TCR by iNKT cell ligands expressed on various pathogens. Second is indirect activation of iNKT cells is through other immune cells and is due to the cytokine milieu and toll-like receptors (TLRs) agonists. In the first type of response, iNKT cells directly recognize glycolipids and lipoproteins, highly abundant in cell walls of many pathogens. These include glycosphingolipid in Gram-negative bacteria *Sphingomonas*, diacylglycerol in *Borrelia burgdorferi*, phosphatidylinositol mannoside in *Mycobacterium tuberculosis*, and glycosphingophospholipid in the protozoa *Leishmania donovani* (13). In the second type of response, iNKT cells are activated through macrophages during microbial infections. When infected, antigen-presenting cells (APCs) recognize bacterial signals *via* innate receptors, such as TLRs of macrophages, e.g., TLR4, TLR7, and TLR9. These allow macrophages to produce inflammatory cytokines, e.g., IL-12, which activate iNKT cells (14).

### Two Types of iNKT Based Vaccines by Focusing on iNKT-Licensed B Cells and iNKT-Licensed DCs to Induce Humoral Immunity

**Figure 1:**
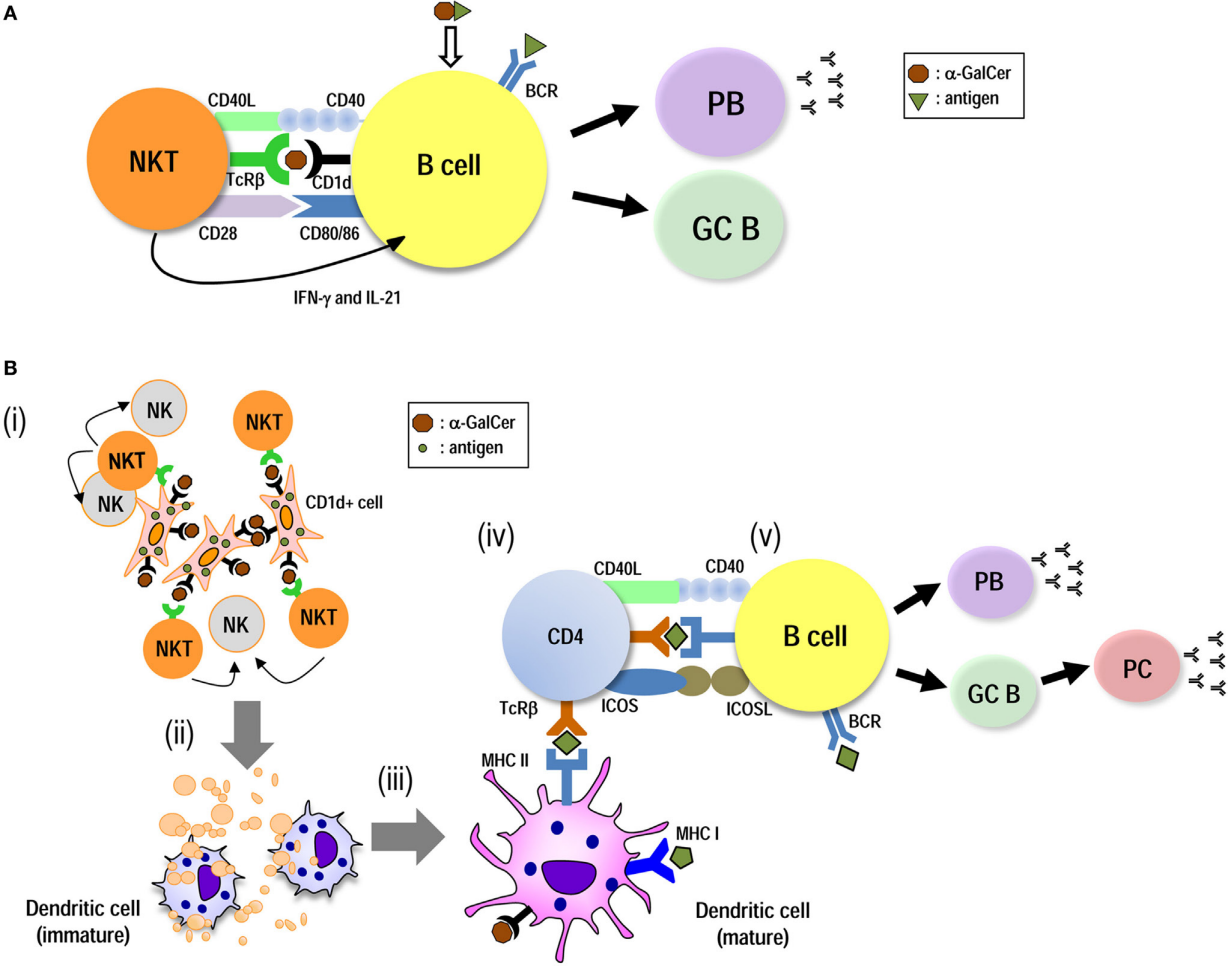
Induction of humoral immunity by utilizing iNKT-licensed B cells and iNKT-licensed dendritic cells (DCs). iNKT cells can provide both direct **(A)** and DC-mediated **(B)** help for antigen-specific B cell responses. **(A)** Innate iNKTfh cells recognize glycolipid antigen-CD1d on B cells and directly provide helper signals. These initiate plasmablast (PB) expansion and germinal center (GC) formation, leading to the primary class-switched antibody (Ab) production. **(B)** Antigen-expressing artificial adjuvant vector cells [e.g., artificial adjuvant vector cells (aAVC)-hemagglutinin (HA)] are comprised of the CD1d–α-GalCer complex on the surface of the aAVC and HA are expressed in the cytosol. (i) Administration of aAVC-HA initially stimulates iNKT cells. (ii) The aAVC-HA is killed by iNKT cells and NK cells and then HA tumor antigen released from them can be captured by endogenous CD11c^+^ DCs. (iii) The CD11c^+^ DCs then undergo iNKT cell-induced maturation. (iv) The activated DCs can then induce an HA-specific CD4^+^ T cell response. Thus, the CD11c^+^ DCs *in situ* are able to present HA antigen derived from phagocytosed aAVC-HA to CD4^+^ T cells. (v) On the other hand, B cells also capture HA antigen. B cells can then be stimulated by antigen-specific CD4^+^ T cells, resulting in PB expansion, GC formation, and long-term Ab production.

B cell response is generally defined as being cognate helper T (Th), namely Tfh-dependent (TD) or Tfh-independent (TI) (3). In terms of iNKT-mediated vaccines directed toward B cell responses, there are two strategies using “iNKT mediated-DC therapy” or “iNKT mediated-B cell therapy” (Figure 1). For the induction of humoral responses, iNKT cell-licensed DCs (non-cognate help) can induce Tfh cells, resulting in long-lasting antibody responses. On the other hand, iNKT cell-licensed B cells (cognate help) generally result in rapid and robust, but short-term responses. However, under some conditions, iNKT cell and B cell interactions seem to be more flexible and may be dependent on the experimental models. These are further discussed below.

#### Vaccines Using iNKT-Licensed B Cell Responses to Induce iNKTfh-Mediated Humoral Immunity

A lipid antigen component, e.g., a hapten or a lipid antigen conjugated to CD1d-binding glycolipids, can induce T-independent Ab production (15, 16). In addition to lipid antigens, a coadministration of T-dependent protein antigen plus α-GalCer, certain protein antigen conjugates, including protein–α-GalCer conjugates or protein incorporated into α-GalCer-liposomes, have been introduced as vaccine formulations for cognate iNKT help (15).

When such immunogens are taken up *via* antigen-specific BCRs, they promote extensive BCR cross-linking and enhance BCR internalization. Simultaneously, the iNKT cell ligand from the immunogen is loaded on CD1d inside of B cells and then expressed on the cell surface, resulting in a strong cognate iNKT–B cell interaction (16–19) (Figure 1A). Without incorporating the iNKT cell ligand, a T-independent response usually elicits only short-lived IgM antibody without germinal center (GC) formation, affinity maturation, and class switch recombination (18). However, with an iNKT cell ligand, even as a T-independent response, iNKT cells are converted to iNKTfh cells in a BCL6-dependent manner. iNKT cells help B cells to proliferate and differentiate into extrafollicular plasma cells. They also robustly and rapidly induce GC formation, low-affinity antibody maturation, secretion of high titers of specific IgM, and early class-switched antibodies. On the other hand, this humoral immunity is short-lived and does not form a memory response (20–22).

In studies of the mechanism of iNKT-licensed B cell-dependent Ab production by lipid- and/or protein-conjugated complexes, several factors have turned out to be essential. Since iNKT cells in steady state are usually located in the marginal zone in spleen or interfollicular or medulla areas in LNs (23, 24), iNKT cells can more easily be activated by contacting marginal zone B cells than by follicular B cells after administration of α-GalCer (16, 21). The cognate help of iNKT cells for B cells presenting CD1d-lipid is dependent on IFN-γ, IL-21, CD40/CD40L, and B7-1/2 (16, 20, 21). SAP expression in iNKT cells plays a key role in the cognate help for antigen-specific B cells, although this is not the case for non-cognate helper functions (25).

In terms of vaccine strategy, several types of vaccines have been described including T-dependent protein antigens combined with α-GalCer and these in nanoparticle liposome formulations (20–22). These showed the direct interaction between B cells and iNKT cells in response to T-dependent antigen, however, these strategies did not bring about high or long duration Ab production (20–22). On the other hand, other investigators have used different conditions and could demonstrate long-term responses. Lang et al. showed that immunization with T-dependent protein antigen together with α-GalCer in the presence of Th cells resulted in long-lasting Ab titers (26–28). A model using coadministration of NP-KLH and α-GalCer elicits Ig class-switched Ab production and requires Th cells and CD1d on B cells, but not on DCs. Here, BAFF- and APRIL-secreting NKT cells play a role in plasma cell longevity (27, 28). Also, liposomal nanoparticles displaying both lipid and polysaccharide antigens induce interactions with DCs for iNKT cell activation and then can elicit long-term B cell memory (29). Thus, the successful induction of iNKT cell-licensed B cells *in situ* demands three signals (Figure 1), BCR cross-linking (signal 1), costimulatory molecules, such as CD28-CD80/CD86, SLAM-family receptor signaling, and CD40-CD40L (signal 2), and inflammatory cytokines (IFN-γ and IL-21) (signal 3).

#### Vaccines Using iNKT-Licensed DC Responses to Induce Tfh-Mediated Humoral Immunity

We previously showed that coadministration of OVA protein antigen plus α-GalCer elicited both CD4^+^ and CD8^+^ T cell responses *via* DC maturation (30). Similar responses have been demonstrated in some cancer and infectious disease models (31–33). We elucidated the mechanism of iNKT-licensed DCs for T cell response; activated iNKT cells promote DC maturation *via* CD40/40L signaling and cytokines (IFN-γ and TNF-α). DCs *in situ* capture the protein antigen and α-GalCer-simultaneously, then present α-GalCer on CD1d to iNKT cells and the peptide on MHC class II to CD4^+^ T cells or class I to CD8^+^ T cells (34, 35). The antigen-specific CD4^+^ Tfh cells that are derived from the helper CD4^+^ T cells stimulate antigen-specific B cells that had already taken up the protein *via* the BCR and presented peptide in MHC class II (18, 36) (Figure 1B). Therefore, expression of both CD1d and MHC class II on DCs and MHC, II but not CD1d on B cells is essential for Ab production (37). Such a vaccine elicits a strong Ab response, i.e., characterized by GC formation, high affinity maturation, primary class-switched Ab, plasma cells, and memory B cells (37, 38).

In the steady state, DCs express CD80/86 to some extent. After activation by iNKT cells, expression of costimulatory molecules, i.e., CD80/86 and CCR7, on DCs is promptly upregulated. The mature DCs are key players for priming CD4^+^ Tfh cells (3). When focused on DC subsets, we and others showed that the XCR1^−^ DC (CD8^−^ DC) subset is superior to the CD8^+^ DC subset for CD4^+^ Tfh cell priming (39). Shin et al. demonstrated this by using mAb to identify specific DC subsets, i.e., anti-DEC205 for the CD8^+^ DC subset or anti-DCIR2 antibodies for the CD8^−^ DC subset, and also showed that the CD8^−^ DCs subset is superior to the CD8^+^ DC subset because of its dominant ICOSL and OX40L expression (40).

The location of DC and iNKT cells before and after iNKT cell activation has recently been clarified. As discussed previously, in the steady state, iNKT cells are localized in the marginal zone (MZ) of the spleen or medulla in LN (41), whereas XCR1^−^ DCs (CD8^−^ DC or 33D1^+^ DCs) are localized in the bridging channel (BC), a unique region of the spleen that spans the interface between the red pulp (RP) and white pulp (WP) and XCR1^+^ DCs reside in the MZ. After i.v. immunization with antigens and/or adjuvants, the majority of both DC subsets migrate into the WP, but XCR1^+^ DCs preferentially go to the CD8^+^ T cell area and XCR1^−^ DCs prefer to go to the CD4^+^ T cell area (42, 43). After activation by α-GalCer or ligand-containing cells, activated iNKT cells accumulate in the MZ or BC and can be in close contact with DCs that have already taken up antigen and α-GalCer (44, 45). iNKT-licensed DCs then relocate to each T cell area.

Germinal center formation in secondary lymphoid organs is considered key for inducing better Ig class switching, somatic mutation, affinity maturation, and long-lasting Ab responses (46). In addition to Bcl6^fl/fl^CD4-cre mice, LTα^−/−^ mice and Lyn^−/−^ mice are deficient in GC formation. When LTα^−/−^ mice and Lyn^−/−^ mice are immunized with antigen plus adjuvant, they respond with long-lasting Ab production, probably due to the generation of long-lived plasmablasts (19, 47–51). However, judging from the data using Bcl6^fl/fl^CD4-cre mice, the formation of GC is essential for iNKT cell-mediated Ab production. When NKTfh and Tfh cells are compared, iNKTfh cells induce earlier GC formation, but it is more short-lived compared to GCs induced by Tfh cells (4). On the other hand, Tfh cells help in the induction of mature GCs better than iNKTfh cells (4). The expression of Bcl6 by Th cells is apparently crucial for efficient Ab production. Thus, iNKT cell-licensed DCs induce antigen-specific CD4^+^ Tfh cells and drive them into the B cell zone (Figure 1B). Then, Tfh cells are engaged in cognate interactions with B cells, resulting in the formation of early GCs and also leading to the long-lasting production of antigen-specific Abs.

The development of effective vaccines is a critical need. As discussed above, immunization by coadministration of protein antigen together with an iNKT cell ligand clearly generates potent Ab production. Traditional immunization protocols usually require a high dose of protein, e.g., 100–500 µg OVA protein per mouse is typically injected (15) yet, even so, Ab production is not impressively high (52). We have reported the artificial adjuvant vector cell (aAVC) system as an efficient vaccine strategy that can potently induce innate and adaptive immunity and this will be discussed in detail in the next section. But, to summarize this section on the induction of antigen-specific Tfh cells and Ab, effective iNKT cell-licensed DCs *in situ* are required (30, 34, 35, 53). The DCs require three factors: (i) expression of the appropriate antigen peptide–MHC complex, (ii) upregulation of costimulatory and chemokine molecules, including CD80/CD86, ICOSL, and OX40L, and (iii) production of inflammatory cytokines and chemokines, such as IL-6, IL-12, and CCL17.

#### An Efficient Strategy Using iNKT-Licensed DCs to Induce Humoral Immunity

We have established the aAVC system, comprised of a CD1d^+^ cellular vaccine incorporating foreign protein antigen plus an iNKT cell glycolipid antigen. We chose “adjuvant vector cell” as the name for this cellular vaccine to describe the fact that the “vector like cells” deliver the antigen as well as an iNKT cell adjuvant to host DCs. We used NIH3T3 cells for mouse and HEK293 cells for humans as vector cells (54, 55). These cells are co-transfected with CD1d and antigen mRNA, and then loaded with α-GalCer for use (54–56). The aAVC, therefore, express the α-GalCer–CD1d complex on their surface and antigen protein intracellularly. The aAVCs directly activate iNKT cells *via* the α-GalCer ligand, and iNKT cells producing IFN-γ can then simultaneously activate NK cells. The combination of innate killer iNKT/NK cells capable of producing IFN-γ then eliminates the adjuvant vector cells, which is not syngeneic with the recipient. Subsequently, the killed aAVC are taken up by DCs (CD8^+^ or XCR1^+^ DCs) *in situ*, thereby several immunogenic features of DCs are engaged. The aAVC captured by DCs in lung, liver, and spleen undergo maturation due to interaction with CD40L on iNKT cells and then produce inflammatory cytokines. The aAVC vaccine can efficiently generate antigen-specific CD8^+^ T cells and memory T cells (36). Interestingly, in aAVC-vaccinated mice, antigen-specific CD4^+^ Tfh primed by XCR1^−^ DCs and GC were both generated, resulting in induction of long-term Ab production (39). Killed aAVCs are phagocytosed, mainly by XCR1^+^ DC (CD8^+^ DC) cells and, presumably, the separated protein antigen and α-GalCer are endocytosed simultaneously by the XCR1^−^ DC subset. MHC II^−/−^ mice do not have CD4^+^ T cells but do have iNKT cells. Immunization with aAVC-OVA elicited no detectable specific Ab response in these mice (39), indicating that iNKT cell-licensed DC strategies require MHC II for CD4^+^ T cell-mediated humoral immunity. Although both Tfh and iNKTfh co-exist in the aAVC-mouse models, Tfh cells are superior to iNKTfh cells for inducing Ab production (39).

## Application of an iNKT Cell-Triggered DC-Designed Vaccine “Artificial Adjuvant Vector Cell” for Influenza Infection

Influenza virus is a member of the orthomyxoviridae family that contains a segmented negative-sense single-stranded RNA genome (57). Influenza infection is a major global health problem, which is initially caused by viral infection of the respiratory tract. Upon viral infection, iNKT cells in the follicular areas of LNs facilitate IL-4 signaling to B cells which triggers the seeding of GC cells and B cell immunity (23). However, to provide sufficient protection against influenza virus in humans, both adaptive T cell and Ab-producing B cells need to be established and maintained as immunological memory.

Immunity against influenza virus is largely mediated by neutralizing antibodies that target the major surface glycoprotein hemagglutinin (HA) (58), in particular, the immunodominant head region of HA, or the viral neuraminidase (NA) (59). Preexisting neutralizing Ab, rather than the recall of virus-specific CTL, is thought to account for memory anti-viral protection. Unfortunately, however, the antiviral antibodies generated by immunization or natural infection are only effective against a limited number of viral strains.

**Figure 2:**
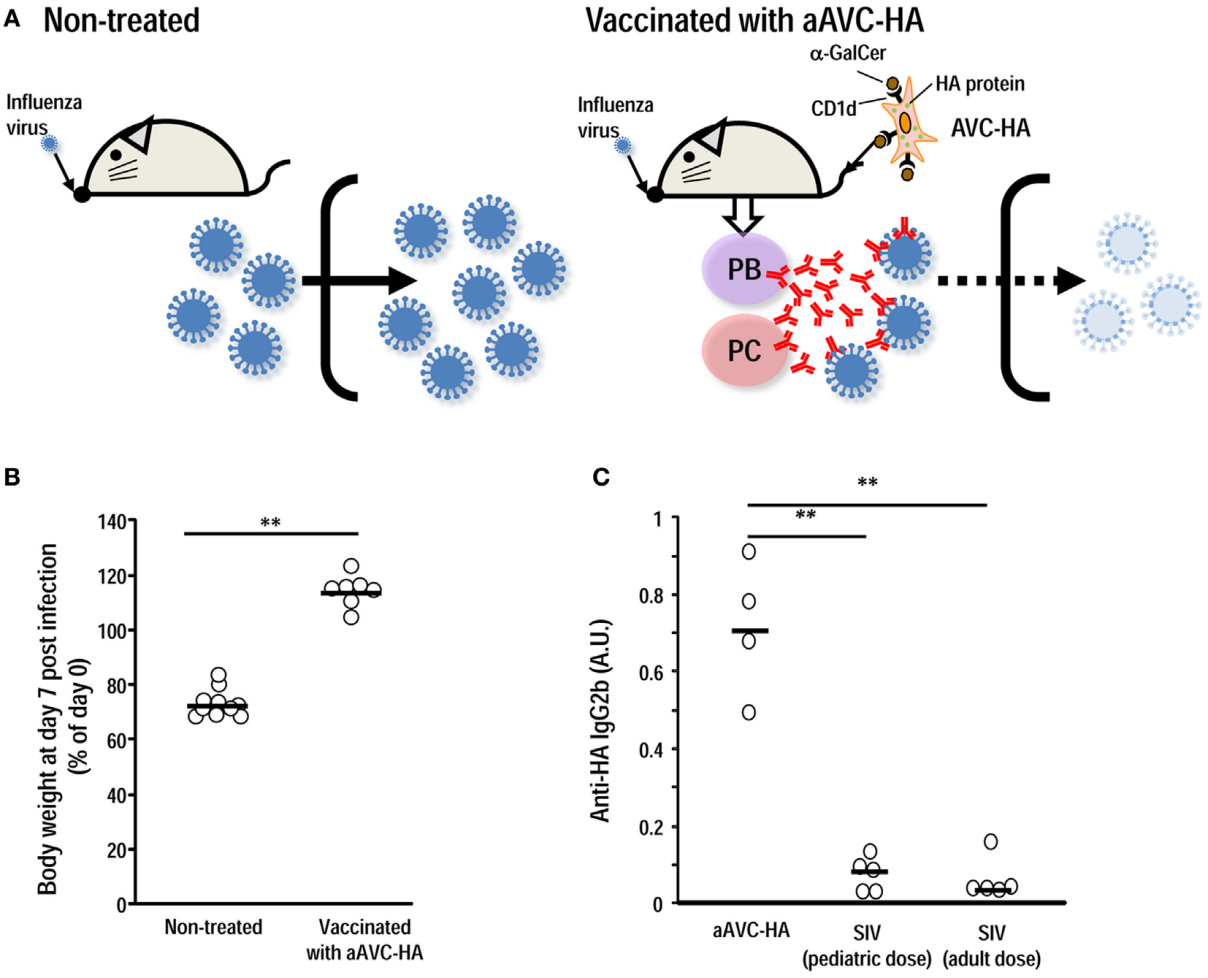
Vaccination with aAVC-HA protects against influenza virus infection. **(A)** Mice were initially vaccinated with 5 × 10^5^ aAVC-HA. Two weeks later, non-treated and the aAVC-HA vaccinated mice were challenged with a lethal dose of PR8 influenza virus to assess production of virus-neutralizing antibody. **(B)** Non-treated and vaccinated mice were evaluated for weight loss a week after an infection. **(C)** HA-specific antibody in the serum was assessed by ELISA 2 weeks after administration of three types of vaccines to C57BL/6 mice, two standard doses of the standard influenza vaccine (0.75 µg/kg, the human pediatric dose, and 0.3 µg/kg, the human adult dose) or the aAVC-HA (mean ± SEM, *n* = 4–7) **P < 0.01.

Several studies have addressed the possibility of using a combined vaccine approach, i.e., coadministration of inactivated influenza virus (IIV) together with α-GalCer, to enhance protective efficacy *via* subcutaneous or intranasal administration (60–62). We established CD1d^+^ HA mRNA-transfected cells loaded with α-GalCer (aAVC-HA) and demonstrated that this is a more efficient strategy for generating antigen-specific Ab production than coadministration of antigen plus α-GalCer (Figure 2) (39). The efficacy of aAVC appears to depend on the GC and Tfh. We could easily modify the HA protein, depending on the circulating virus strain, and the manufacturing process for this vaccine has a much shorter timeframe than others, e.g., egg-derived vaccines, which would be especially valuable during a flu pandemic. The aAVC vaccine thus holds great promise as a potential broad spectrum prophylactic or therapeutic agent and for the development of a universal influenza or other viral vaccine (39). As a future study, if the optimal stem region antigen were incorporated into an iNKT cell-mediated vaccine, a universal HA stem antigen-expressing aAVC could be developed.

## Conclusion

iNKT cells play an immunomodulatory role during immune responses. To utilize this capacity as an adjuvant in terms of novel vaccine strategies, it is essential to understand the interaction of iNKT cells with APCs in the host. We here summarized details of the relationship of iNKT cell-triggered B cells and DCs with Ab production and compared them in terms of vaccine development. Now we need to consider the future directions and challenges in translating these findings from experimental data obtained from mice to use in the clinic.

## Author Contributions

SF and KS conceptualized, wrote, and edited the manuscript. SY and YS wrote and edited the manuscript.

## Conflict of Interest Statement

The authors declare that the research was conducted in the absence of any commercial or financial relationships that could be construed as a potential conflict of interest.
